# Shedding light on the neonatal brain: probing cerebral hemodynamics by diffuse optical spectroscopic methods

**DOI:** 10.1038/s41598-017-15995-1

**Published:** 2017-11-17

**Authors:** Parisa Farzam, Erin M. Buckley, Pei-Yi Lin, Katherine Hagan, P. Ellen Grant, Terrie Eleanor Inder, Stefan A. Carp, Maria Angela Franceschini

**Affiliations:** 1 Athinoula A. Martinos Center for Biomedical Imaging, Massachusetts General Hospital, Harvard Medical School, Boston, MA 02129 USA; 2 Georgia Institute of Technology, Atlanta, GA 30322 USA; 3 Fetal-Neonatal Neuroimaging and Developmental Science Center, Division of Newborn Medicine, Boston Children’s Hospital, Harvard Medical School, Boston, MA 02115 USA; 4 Department of Pediatric Newborn Medicine, Brigham and Women’s Hospital, Harvard Medical School, Boston, MA 02115 USA

## Abstract

Investigating the cerebral physiology of healthy term newborns’ brains is important for better understanding perinatal brain injuries, of which the most common etiologies are hypoxia and ischemia. Hence, cerebral blood flow and cerebral oxygenation are important biomarkers of brain health. In this study, we employed a hybrid diffuse optical system consisting of diffuse correlation spectroscopy (DCS) and frequency-domain near infrared spectroscopy (FDNIRS) to measure hemoglobin concentration, oxygen saturation, and indices of cerebral blood flow and metabolism. We measured 30 term infants to assess the optical and physiological characteristics of the healthy neonatal brain in the frontal, temporal, and parietal lobes. We observed higher metabolism in the right hemisphere compared to the left and a positive correlation between gestational age and the level of cerebral hemoglobin concentration, blood volume, and oxygen saturation. Moreover, we observed higher cerebral blood flow and lower oxygen saturation in females compared to males. The delayed maturation in males and the sexual dimorphism in cerebral hemodynamics may explain why males are more vulnerable to perinatal brain injuries than females.

## Introduction

Although neonatal brain comprises only 10% of the body weight, it accounts for more than 50% of whole body basal metabolic rate, which highlights the importance of the blood flow and energy supply to the brain^1^. Deficiencies in oxygen content (hypoxia) or reduced blood flow (ischemia) in the brain are the major causes of mortality in newborns. Furthermore, lack of oxygen delivery to the brain may cause seizures, cognitive impairment, and other neurological disabilities^2–4^. Thus, cerebral blood flow (CBF) and cerebral oxygenation (SO_2_) are important biomarkers of brain health and function. To understand pathological mechanisms and early biomarkers of perinatal brain injury, it is critical to study healthy brain development. Although techniques that quantify CBF and/or SO_2_, such as magnetic resonance imaging (MRI), positron emission tomography (PET), and Doppler ultrasound are becoming more common, they are not suitable for continuous monitoring over time. Moreover, due to complications in data acquisition, these techniques are usually restricted to infants with clinical symptoms^5,6^ and are not commonly used in healthy term infants. Considering the importance of assessment of cerebral blood flow and oxygenation, in this study we employed diffuse optical spectroscopy techniques for noninvasive estimation of cerebral blood flow, total hemoglobin concentration (HbT), and oxygen saturation in healthy term newborn infants^7,8^. We applied a hybrid device consisting of a frequency-domain near-infrared spectroscopy (FDNIRS) for measurement of hemoglobin concentration and oxygen saturation^9^ in tandem with diffuse correlation spectroscopy (DCS) for measurement of an index of cerebral blood flow (CBF_i_)^10–12^. The unit of this index is [cm^2^/sec], which is different from conventionally reported blood flow units. However, DCS measurements agree well with the CBF values measured by other modalities including perfusion MRI^13,14^, Doppler ultrasound^15,16^, contrast-enhanced computed tomography^17,18^, and fluorescent microspheres^19^. Concurrent assessment of blood flow and oxygen saturation can provide information regarding an index of cerebral metabolic rate of oxygen (CMRO_2i_)^20,21^, an important marker of brain health since the brain is metabolically one of the most active organs in the body and its energy supply is based on aerobic metabolism, indicating the importance of oxidative metabolism in the brain and its monitoring^22–31^.

We have performed noninvasive optical measurements in 30 term infants to determine the optical (absorption and scattering coefficients) and physiological (blood flow, hemoglobin concentration, and oxygen saturation) characteristics of healthy human brain in the first days after birth. We have obtained the distribution of the measured parameters for this healthy population and we have observed higher CBF_i_ and CMRO_2i_ in the right hemisphere compared to the left hemisphere. Moreover, this study reveals significant sexual dimorphism in measured hemodynamic parameters. Although sexual differences in brain have been examined from neurobehavioral and neurodevelopmental perspectives^32–34^, relatively few studies have focused on cerebral hemodynamics differences between males and females. In this study, we have focused on early physiological sexual dimorphism in newborns’ brains. We have observed that in healthy neonates, girls tend to have lower oxygen saturation and higher blood flow than boys. These findings may be part of the reason for which newborn males are more vulnerable to white matter injury, hypoxic-ischemic encephalopathy, and cerebral palsy than newborn females^35–37^.

## Results

### Optical and physiological characteristics of healthy brain after birth

**Figure 1:**
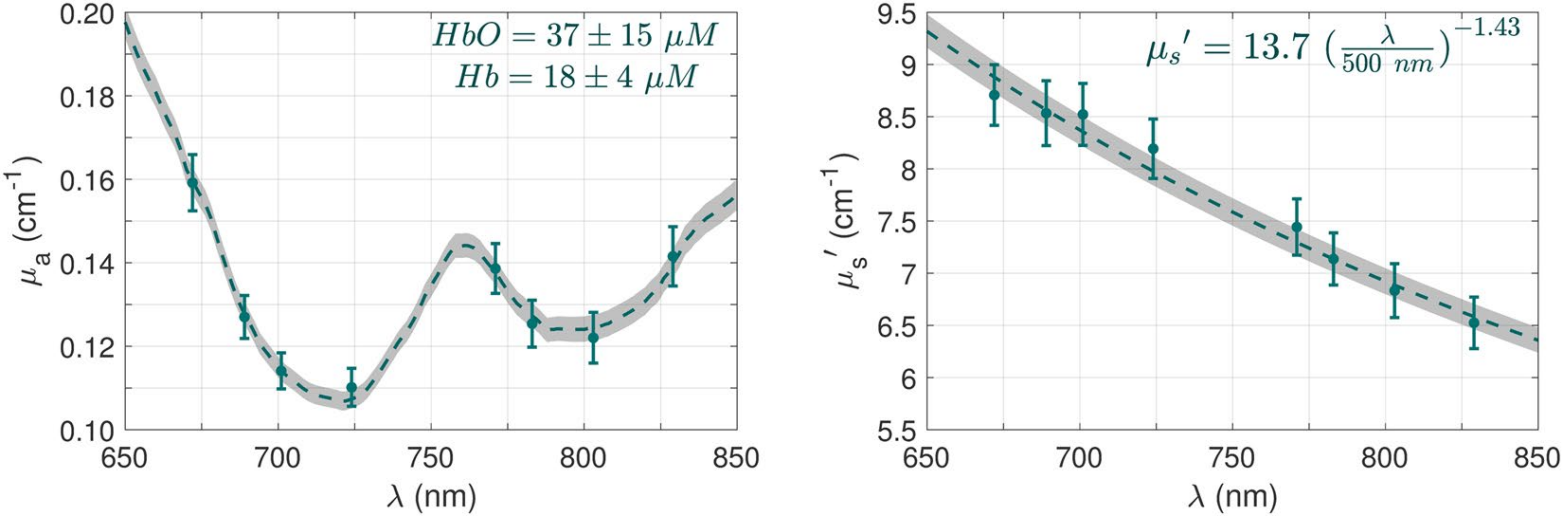
The measured absorption coefficient (*μ*_*a*_) and reduced scattering coefficient $({\mu }_{s}{\rm{^{\prime} }})$ of healthy newborns’ brains and their fitted spectrum. The green error bars indicate the mean and 95% confidence interval over all babies and locations. The dashed green line is the mean of fitted spectrum and the gray shaded area indicates the 95% confidence interval of the mean.

Figure 1 shows the grand average of optical properties, i.e., the absorption coefficient (*μ*_*a*_) and reduced scattering coefficient (${\mu }_{s}{\rm{^{\prime} }}$), across all the measured infants and brain locations. Error bars represent the 95% confidence intervals. The dashed green line is the mean of the fitted spectrum and the gray shaded area indicates the 95% confidence interval of the mean. The resulting average oxy- and deoxy-hemoglobin concentrations are 37 ± 15 (μM) and 18 ± 4 (μM), respectively. For the scattering, the average number density (a) is 13.7 ± 3.5 and the average effective particle size (b) is 1.43 ± 0.43, for the relationship derived from the Mie model $({\mu }_{s}{\rm{^{\prime} }}(\lambda )=a{(\frac{\lambda }{{\lambda }_{ref}})}^{-b},\,{\lambda }_{ref}=500\,{\rm{n}}{\rm{m}}).$

**Figure 2:**
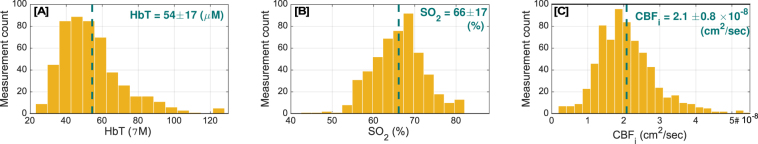
The distribution of the measured hemodynamic parameters and the mean standard deviation of the distribution. Histogram of [**A**] total hemoglobin concentration (HbT), [**B**] oxygen saturation (SO_2_), and [**C**] cerebral blood flow index (CBF_i_).

The histogram of the measured hemodynamic parameters and the mean ± standard deviation of the distribution across all infants and brain locations measured are presented in Fig. 2. Panel [A] is the distribution of the total hemoglobin concentration with the average value of 54 ± 17 (μM). Panel [B] shows the histogram of oxygen saturation with the average value of 66 ± 17%. Panel [C] presents the distribution of DCS-measured cerebral blood flow index with the average of 2.1 ± 0.8 × 10^−8^ (cm^2^/sec).

When comparing measured locations, we observed hemodynamic differences between the left and right hemispheres. The average CBF_i_ and CMRO_2i_ in the right hemisphere are, respectively, 15.2% (P = 0.01) and 15.4% (P = 0.01) higher than in the left hemisphere. There was no significant difference in oxygen saturation and hemoglobin concentration level between left and right.

### Sexual dimorphism in cerebral hemodynamics

**Figure 3:**
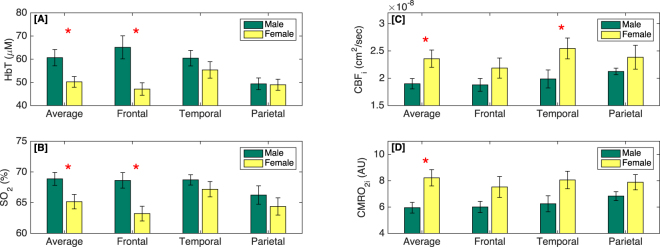
The sexual differences in cerebral hemodynamics represented with bar graphs, reporting the mean values and standard errors across infants. The green bars indicate males; yellow bars, females; and the red stars indicate the locations where there is statistically significant difference between males and females. The x-axis shows the site of measurement: the average over all locations, frontal, temporal, and parietal lobes. [**A**] Total hemoglobin concentration (HbT). [**B**] Oxygen saturation (SO_2_). [**C**] Cerebral blood flow index (CBF_i_). [**D**] Cerebral oxygen metabolism index (CMRO_2i_).

We found that the cerebral hemodynamics differ between male and female newborns. This sexual dimorphism is shown in Fig. 3 with bar graphs (mean values and standard errors). In all panels, x-axis indicates the site of measurement: average (average of all locations), frontal, temporal, and parietal lobes. Although measurements in the left and right hemispheres are averaged together, the significant side differences are investigated considering side as a random variable in the linear mixed effects (LME) fitting. Red stars indicate statistically significant differences between males and females. Figure 3, panel [A] shows that males have higher HbT than females in all locations but the difference is statistically significant only in the frontal lobe (P = 0.028) and in the average of all locations (P = 0.009). Panel [B] shows that the oxygen saturation of males is generally higher than that of females and the difference is significant in the average (P = 0.034) and frontal lobe (P = 0.006). Panel [C] shows that females tend to have higher cerebral blood flow index (CBF_i_) than males and the difference is significant in the average (P = 0.048) and the temporal lobe (P = 0.049). Similarly, the cerebral oxygen metabolism (CMRO_2i_) in females tends to have higher values than in males, panel [D]. The difference is statistically significant in the average of all locations (P = 0.004).

### The effect of gestational age on cerebral hemodynamics

**Figure 4:**
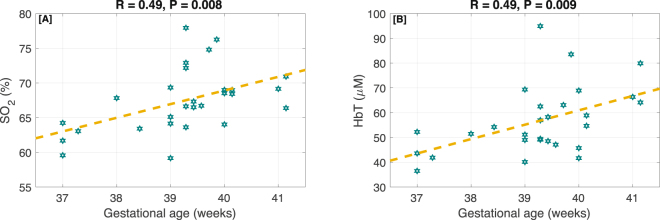
The correlation between gestational age and [**A**] SO_2_, [**B**] HbT. The dotted lines show the best linear fits.

Figure 4 shows the correlation between gestational age (GA) and measured HbT and SO_2_. In panel [A], it is observed that newborns with higher GA tend to have higher total oxygen saturation. The Pearson correlation coefficient, R, is 0.49 (P = 0.008). Similarly, panel [B] shows that GA has a direct relationship with the total hemoglobin concentration (R = 0.49 and P = 0.009). This relationship was still statistically significant after accounting for sex, brain hemisphere, and cortical region (P = 0.002 for SO_2_ and P = 0.002 for HbT). To cancel out the effect of sex, brain hemisphere, and cortical region we fit at LME model with gestational age as the fixed effect and considering subjects, brain sides, lobes, and sex as the random effects. CBF_i_ and CMRO_2i_ did not show any statistically significant trend with GA.

## Discussion

In his work, diffuse optical spectroscopy techniques are used to characterize the optical (absorption and reduced scattering coefficients) and physiological (HbT, SO_2_, and CBF_i_) properties of the healthy human newborn brain over the left and right frontal, temporal, and parietal lobes. We have developed a combined FDNIRS-DCS instrument for simultaneous measurement of blood flow, oxygen saturation, and hemoglobin concentration. This device benefits from innovation and novelty in design, construction, and user interface. It is the first truly concurrent measurement of DCS and FDNIRS due to the use of higher wavelengths for DCS and optical low pass filters in front of the FDNIRS detectors to prevent the FDNIRS detectors from seeing the powerful DCS laser light, which eliminates the need for sequential measurements. Moreover, we have developed live feedback software that inspects the signal and fitting quality and warns the user in case of a low-quality measurement (data quality assessment described below). Hence, the user can repeat the measurement after slightly relocating the probe to prevent any imperfect contact of the probe with the skin, hair under the fibers, etc., to have a better chance of recording high-quality data over all the desired locations. These features enable us to obtain data with sufficient signal-to-noise ratio with each measurement. The optical probe consists of source-detector separations between 1.5 and 3 cm and the penetration depth has a direct relationship with the source-detector distance^38^. As a rule of thumb, the mean light penetration depth in the reflection geometry is in the order of one half of the optode distance, which in case of the current probe geometry is sufficient to measure the superficial brain cortex in newborns^39^. The population-averaged spectra of the fitted absorption and reduced scattering coefficients are presented in Fig. 1 and they are in good agreement with the literature values on healthy infants reported by Demel *et al*.^40^. The average of the number density (a = 13.7 ± 3.5 cm^−1^) and effective particle size (b = 1.43 ± 0.43) derived by the Mie model is in the range of brain values listed by Steven Jacques^41^ for brain tissue. The distributions of the measured cerebral hemodynamic parameters are presented in Fig. 2. The average values of total hemoglobin concentration and oxygen saturation are 54 ± 17 μM and 66 ± 17%, respectively. These values are consistent with the values found using an older-generation FDNIRS device on 22 term newborns^25^ and another study from our lab with 13 health neonates^24^. Moreover, our results lie within the range of reported values from several relevant studies with a mixed population of term, preterm, healthy, and unhealthy infants. In a study^8^ with a mixed preterm and term population, the reported HbT and SO_2_ at the first week of life are 52 μM and 67%, respectively, which matches very well with the measured values in this study. The oxygenation values of 66–70% for healthy and unhealthy term neonates^24,25^ and 70% for a mixture of healthy, unhealthy, term, and preterm^42^ are also in good agreement with our current results. On the other hand, the studies in premature infants report lower values in general. For instance, Roche-Labarbe *et al*.^43^ reported HbT = 40 ± 7 μM and SO_2_ = 73 ± 4%. In another study on preterm neonates^44^, HbT = 39.4 μM and SO_2_ = 62%, which are again lower than the values in the current study.

The right-skewed blood flow index distribution is similar to the previously measured distribution of blood flow index^45^. Comparing our measured cerebral blood flow index, CBF_i_ = (2.1 ± 0.8) × 10^−8^ cm^2^/sec (see Fig. 2C), to the values from the healthy control group in Dehaes *et al*.’s study^24^, we see that they reported a higher value: CBF_i_ ≈ 3.5 ×10^−8^ cm^2^/sec. The difference is due to their assumption of a fixed reduced scattering coefficient (${\mu }_{s}{\rm{^{\prime} }}$ (785 nm) = 5 cm^−1^) in the DCS analysis, which is lower than the value measured and used in this study (${\mu }_{s}{\rm{^{\prime} }}$ (850 nm) = 6.4 cm^−1^). It has been known that underestimation of ${\mu }_{s}{\rm{^{\prime} }}$ leads to overestimation of CBF_i_^46^.

Similar to a previous finding from our lab (see ref.^30^), we observed significantly higher CBF_i_ and CMRO_2i_ in the right hemisphere compared to the left while there was no significant difference between left and right in the level of SO_2_ and HbT. The higher blood flow and metabolism in the right side might be due to the delay in the maturation of the left motor and Broca cortical area to allow for higher plasticity after birth^47^. Moreover, the right hemisphere sustains survival functions, which justifies its earlier development^48^.

In this study, we have observed that cerebral hemodynamic parameters differ between males and females early after birth. This sexual dimorphism is shown in Fig. 3: males systematically tend to have higher HbT than females and the difference is statistically significant in the frontal lobe (P = 0.028) and the average of all lobes (P = 0.009). The oxygen saturation of males is generally higher than that of females and the difference is significant in the frontal lobe (P = 0.006) and the average of all measured lobes (P = 0.034). Instead, females consistently have higher cerebral blood flow index (CBF_i_) than males and the difference is significant in the temporal lobe (P = 0.049) and average of measured lobes (P = 0.048). These differences lead to a higher estimation of the cerebral oxygen metabolism index (CMRO_2i_) in females than in males, panel [D]. Although, the difference is statistically significant in the averaged data (P = 0.004), it might have been driven from differences in blood hemoglobin concentration (HGB). In this study, we do not have the individual HGB values of males and females for accurate estimation of CMRO_2i_. Even though previous studies^49^ in a large population of neonates have shown no significant difference between HGB values of male and female at birth, there might be enough gender differences in our small sample to impact the CMRO_2i_ values.

Our observation of sexual difference in healthy term neonates is similar to that measured in older children and in adults with different modalities^50–55^. As in these studies, the sexual differences that we found cannot be attributed to differences in physiologic or demographic parameters, which are similar in the two groups (Table 1). Interestingly, the sexual difference observed in term neonates, children, and adults are reversed in premature infants. Previously, Baenziger *et al*.^56^ and Stark *et al*.^57^ used intravenous xenon 133 and laser Doppler flowmetry to measure CBF, and showed that, in preterm neonates, the CBF in males is higher than in females. Recently, Lin *et al*.^30^, from our lab, using FDNIRS and DCS confirmed a higher cerebral blood flow in preterm males in most cortical regions. This is the first study reporting the sexual dimorphism in CBF in healthy term newborns. From this study it is not clear at which preterm age the CBF dimorphism is inverted.

**Table 1:** Demographic and physical information of the recruited subjects: mean ± standard deviation.

	Population	C-section	Gestational age (w)	Postnatal age (d)	Weight (Kg)	Length (cm)	Head circumference (cm)
All	30	20 (67%)	39.2 ± 1.2	2.4 ± 1.1	3.49 ± 0.43	50.9 ± 2.5	34.6 ± 1.4
Females	11	07 (64%)	39.1 ± 1.2	2.6 ± 1.5	3.50 ± 0.44	50.8 ± 2.5	34.0 ± 1.2
Males	19	13 (68%)	39.3 ± 1.2	2.4 ± 0.8	3.48 ± 0.43	50.9 ± 2.5	34.9 ± 1.4

Another interesting result from these measurements is the effect of gestational age on oxygen saturation and hemoglobin concentration. As shown in Fig. 4, newborns with higher GA tend to have higher total hemoglobin concentration (R = 0.49 and P = 0.009) and oxygen saturation (R = 0.49 and P = 0.008). There is no statistically significant relationship between GA and CBF_i_ or CMRO_2i_. While this study only investigates term infants and the GA range is very limited (37–42 weeks), the relationship between cerebral oxygenation, and hemoglobin content with GA is statistically significant. These findings are in agreement with a previous study by Roche-Labarbe *et al*.^43^, in which they mentioned a trend of higher HbT and SO_2_ in neonates born with higher gestational age; however, their measured difference did not reach significance levels. Similarly, Ijichi *et al*.^42^ reported a positive correlation between cerebral blood volume and gestational age, yet they report a decrease of cerebral oxygen saturation in neonates with higher gestational age. The discrepancy might be due to the study population, as their study included neonates with clinical concerns such as respiratory disorders. To understand the physiological basis that causes the increased blood hemoglobin concentration, and oxygen saturation in neonates with higher GA, more research on cerebral vasculature network development and individual HGB values are needed, which is not the scope of this paper.

The main limitation of this study is the restricted number of the participated infants due to the sensitive nature of newborns and small time window that they usually spend in the hospital. However, the histograms of the measured parameters show rigid distribution of the data, increasing number of neonates may help us to have higher statistical power. Moreover, in this study, we do not have the individual HGB values since the HGB of a healthy neonate is not measured as a part of standard clinical practice. Therefore, we cannot interpret the reason beyond the gender and age dependencies as they might have been driven by HGB differences.

In conclusion, we have developed a hybrid FDNIRS-DCS instrument and deployed it for noninvasive optical FDNIRS-DCS measurements on 30 healthy term neonates. Furthermore, we characterize the optical and physiological properties of the healthy human brain in the first days after birth. This study reveals significant sexual dimorphism in measured hemodynamic parameters. We observe that newborn girls tend to have lower oxygen saturation and higher blood flow than boys. These findings may help us to better understand the mechanisms that make males more prone to brain injuries than females in the perinatal period^37,58,59^.

## Methods

The study protocol was reviewed and approved by the Institutional Review Board (IRB) for Partners Healthcare, the Partners Human Research Committee (PHRC). The study method was designed and carried out in accordance with PHRC requirements and the regulations that govern human subjects research.

**Figure 5:**
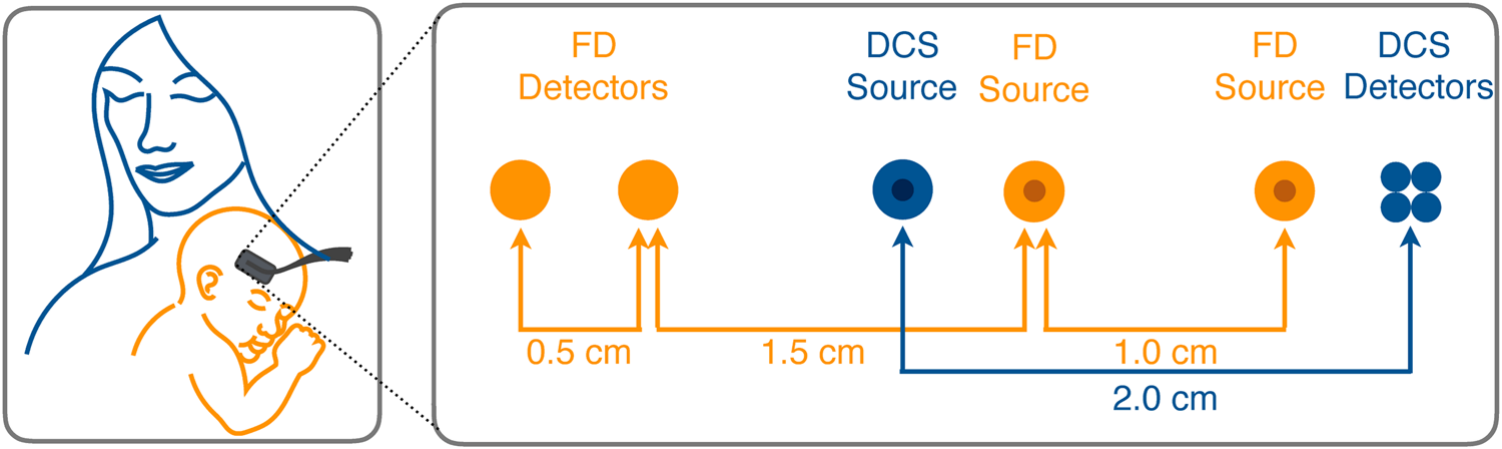
[Left] The probe is gently located on the infant’s head while the infant is resting in the mother’s embrace. [Right] Schematic of the hybrid probe: FDNIRS (orange circles) consists of two sources and two detectors arranged in a way that provides four source-detector separations from 1.5 to 3 cm in steps of half a centimeter. DCS (blue circles) consists of one source and four detectors, all located at a distance of 2 cm from the source.

30 healthy term infants were enrolled from the well baby nursery of Brigham and Women’s Hospital between May/2015 and January/2016. Parents who agreed to participate were asked to read and sign an informed consent form approved by the PHRC. The summary of demographic information of the enrolled infants is listed in Table 1. There is no statistically significant difference between males and females in the reported demographic parameters. In all subjects, we investigated 7 brain cortex regions: right/left temporal, right/left parietal, and right/middle/left frontal. Each location was measured several times by repositioning the probe in a slightly different area to account for the local inhomogeneities beneath the sensor^43^. We measured babies while they were resting in bed or in their mothers’ arms (Fig. 5). All the infants were measured at resting state and if they were moving, feeling irritated, or crying we skipped the measurement. At each measurement the optical sensor was gently held in contact with the skin over the brain region of interest for 10s. In addition, the arterial oxygen saturation (SaO_2_) was recorded using a pulse oximeter at the time of the measurement.

### Optical device and the probe

We employed a hybrid diffuse optical device consisting of DCS and FDNIRS combined in a single probe, as illustrated in Fig. 5.

In frequency domain measurements, light intensity of the source is modulated at the frequency of 110 MHz producing a diffusive wave in the medium oscillating at the same frequency. At a given source-detector separation, both the amplitude and phase of the diffusing wave are measured. The of phase shift increase and intensity decrease over several source-detector separations provide simultaneous determination of *μ*_*a*_ and ${\mu }_{s}{\rm{^{\prime} }}$. DCS uses a long-coherent laser source (CrystaLaser, Reno, NV, USA) at 850 nm. The FDNIRS detectors are protected by filters that block the DCS laser light at 850 nm (20–30 mW), thus enabling concurrent measurement of FDNIRS (10 Hz) and DCS (1.2 Hz). The DCS detectors are not affected by the low-power FDNIRS lasers (2–5 mW). Moreover, the software interface provides online feedback about signal and fitting quality and informs the user about low-quality measurements. The FDNIRS and DCS optical fibers are combined into a single 3D printed probe, which is designed such that all fibers are arranged in a row on black rubber material. All source and detector fibers’ ends are coupled to prisms to shift the emission direction 90° to be perpendicular to the tissue. In series of liquid phantom tests, we did not observe any disturbance in the results due to the prisms of the probe^21^. DCS laser light is delivered to the tissue through a multimode fiber with a core diameter of 200 μm and, at the probe, is diffused by a diffuser to make a larger spot (5 mm diameter), which allows us to use light intensity up to 30 mW without exceeding the ANSI (American National Standards Institute) safety limits for skin illumination. Since the small diameter of single-mode fibers (5.7 μm) at the DCS detectors limits the detected photon count rate, we use 4 photon-counting avalanche photodiodes detectors (Excelitas, Quebec, Canada) in the same location at 2 cm from the source to improve the signal-to-noise ratio. The detectors’ output is fed to a correlator (Correlator.com, NJ, USA) to obtain the autocorrelation functions. Details of the DCS system are discussed in various reviews^60,61^. FDNIRS consists of 16 laser sources (Thorlabs, Inc. NJ, USA) divided into 2 identical groups of 8 lasers emitting in the near infrared (672, 689, 701, 724, 771, 783, 803, 829 nm). Two photomultiplier tubes (R9880U, Hamamatsu, Japan) detect the diffusely scattered light. FDNIRS sources and detectors are coupled to fiber-optic bundles (2.5 mm in diameter) with source-detector distances of 1.5, 2.0, 2.5, and 3.0 cm (Fig. 5).

### Theory and calculations

All the data and statistical analyses of this paper are performed using MATLAB (Mathworks, USA), version R2015a, and Statistics and Machine Learning Toolbox, version 10.0.

### Frequency-domain near-infrared spectroscopy

For the processing of FDNIRS data, we discarded the measurements that have low signal-to-noise ratio (more than 0.05 radian of phase noise). FDNIRS light amplitude and phase shift were calibrated^62^ using a solid phantom with known optical properties provided by ISS, Inc., IL, USA.

The frequency-domain solution of the photon diffusion equation in the semi-infinite geometry was used to fit for the wavelength-dependent optical properties of the tissue (*μ*_*a*_ and ${\mu }_{s}{\rm{^{\prime} }}$)^60^. At each wavelength, we have performed the linear fitting on ln(*AC*.*ρ*^2^) and phase over distance to obtain the slopes, real and imaginary wavenumber of diffusion photon density waves (DPDW), from which one can calculate absorption and reduced scattering coefficients^60^. Data with low-quality fitting (Pearson correlation coefficient, R2 < 0.95) were discarded. The effective particle size and number density, under the model^41,63^, ${\mu }_{s}{\rm{^{\prime} }}(\lambda )=a{(\frac{\lambda }{500{\rm{n}}{\rm{m}}})}^{-b}$, were fitted to the reduced scattering coefficient (${\mu }_{s}{\rm{^{\prime} }}(\lambda )$). In this equation, the wavelength *λ* is normalized by a reference wavelength, 500 nm, resulting in a dimensionless value, which is raised to a power “−b”, scattering power. The wavelength exponent, “b”, is independent of the particle concentration and characterizes the effective particle size and defines the spectral behavior of the scattering coefficient. This term characterizes the wavelength dependence of ${\mu }_{s}{\rm{^{\prime} }}$. The factor “a” depends on the concentration of particles in the media. The measured absorption coefficient (*μ*_*a*_(*λ*)) is related to the different tissue chromophores as ${\mu }_{a}(\lambda ){\sum }_{i=1}^{nc}{\varepsilon }_{i}(\lambda ){c}_{i}$. The sum is over different chromospheres and for biological tissues in the used spectral range the main chromospheres are water, oxy- and deoxyhemoglobin. *ε*_*i*_(*λ*) is the wavelength-dependent extinction coefficient of the i^th^ chromosphere obtained from the literature^64^, and c_i_ is the concentration of the i^th^ chromosphere. The water concentration in the brain is assumed to be 75%^65^ and oxy- and deoxyhemoglobin concentrations are measured (HbO and HbR). The total hemoglobin concentration (HbT) is assumed to be the sum of oxygenated and deoxygenated hemoglobin, i.e., HbT = HbO + HbR and blood oxygen saturation (SO_2_ = HbO/HbT). In both the scattering and absorption spectral fitting, we monitor the goodness of fitting through parameters such as root mean square error, p-value, and correlation coefficient to remove the data points that do not meet the data quality criteria.

### Diffuse correlation spectroscopy

DCS measures temporal fluctuations of speckle patterns on the tissue caused by the motion of scatterers within the tissue. Given that the primary moving scatterers in tissue are red blood cells, we can estimate an index of microvascular blood flow based on the time scale of the speckle fluctuations. We quantify these speckle fluctuations using an intensity autocorrelation function to estimate microvascular blood flow^60,66,67^. The dynamics of the medium can be determined by the measurement of the intensity autocorrelation. The semi-infinite homogeneous medium solution to the correlation diffusion (equation 1) was employed to fit to the measured autocorrelation curves, for the cerebral blood flow index (CBF_i_)^60^.

1 $${G}_{1}(\rho ,\tau )=\frac{3{\mu }_{s}{\rm{^{\prime} }}}{4\pi }[\frac{\exp (-K(\tau )\cdot {r}_{1})}{{r}_{1}}-\frac{\exp (-K(\tau )\cdot {r}_{b})}{{r}_{b}}]$$

Here, *τ* is the delay time ${r}_{1}=\sqrt{{\rho }^{2}+{(1/{\mu }_{s}{\rm{^{\prime} }})}^{2}}$, ${r}_{b}=\sqrt{{\rho }^{2}+{(1/{\mu }_{s}{\rm{^{\prime} }}+2{z}_{b})}^{2}}$, $K(\tau )=\sqrt{3{\mu }_{a}{\mu }_{s}{\rm{^{\prime} }}+6{\mu }_{s}{\rm{^{\prime} }}{\kappa }_{0}^{2}\tau CB{F}_{i}{)}^{2}}$, *z*_*b*_ is the extrapolated zero boundary, and *κ*_0_ is the wavenumber of light in the medium^60^. DCS instrumentation measures the intensity autocorrelation function, while the correlation diffusion equation dictates the behavior of the electric field temporal autocorrelation function. To compare theory with experiment, the normalized intensity autocorrelation function (g_2_) is related to the normalized electric field temporal autocorrelation (g_1_) through the Siegert relation, g_2_(*τ*) = 1 + *β*g_1_^2^(*τ*), where *β* is a constant determined primarily by the coherence of the laser and by the collection optics^60^. The equation has three unknowns: CBF_i_*, μ*_*a*_, and ${\mu }_{s}{\rm{^{\prime} }}$. In this study, for each baby, at each measurement site, we used the measured *μ*_*a*_ from the FDNIRS measurement. For the reduced scattering coefficient, we used a fixed value ${\mu }_{s}{\rm{^{\prime} }}$(850 nm) = 6^.^4 (cm^−1^), which is the average value across all subjects to prevent noise due to fluctuations on measured ${\mu }_{s}{\rm{^{\prime} }}$. In the fitting, the Nelder-Mead derivative-free simplex method (fminsearch), implemented in MATLAB, is used. By combining the FDNIRS and DCS measured parameters, one can estimate the cerebral metabolic rate of oxygenation using Fick’s law: CMRO_2_ = OEF.CBF.CaO_2_, where OEF is the oxygen extraction fraction, CaO_2_ = κ.HGB·SaO_2_ (mL O_2_/dL) is the theoretical maximum oxygen carrying capacity, κ is a constant indicating the amount of oxygen that each gram of hemoglobin can carry (κ = 1.34), and SaO_2_ is the arterial oxygen saturation, which is measured for all neonates using a pulse oximeter. The oxygen extraction fraction estimation is defined as OEF = (SaO_2_ − SvO_2_)/SaO_2_, where SvO_2_ is the venous oxygen saturation. The optically measured tissue oxygen saturation (SO_2_) is a mixture of arterial and venous blood: SO_2_ = (1 − γ)SaO_2_ + γSvO_2_ and by assuming compartmentalization of blood, the relative contributions of each compartment to the optical signal, OEF can be estimated from tissue and arterial oxygenation: OEF = (SaO_2_ − SO_2_)/ (γSaO_2_)^12,21,68^. Thus, CMRO_2_ = κ.HGB.CBF (SaO_2_ − SO_2_). Using the DCS blood flow index and assuming fix HGB, an index of cerebral metabolism can be estimated by CMRO_2i_ = CBF_i_ (SaO_2_ − SO_2_). Since we do not have the HGB for each baby, we cannot accurately compare the cerebral metabolism across subjects but it provides us with an index of metabolism to compare different regions of brain within neonates.

### An example of fitting quality

**Figure 6:**
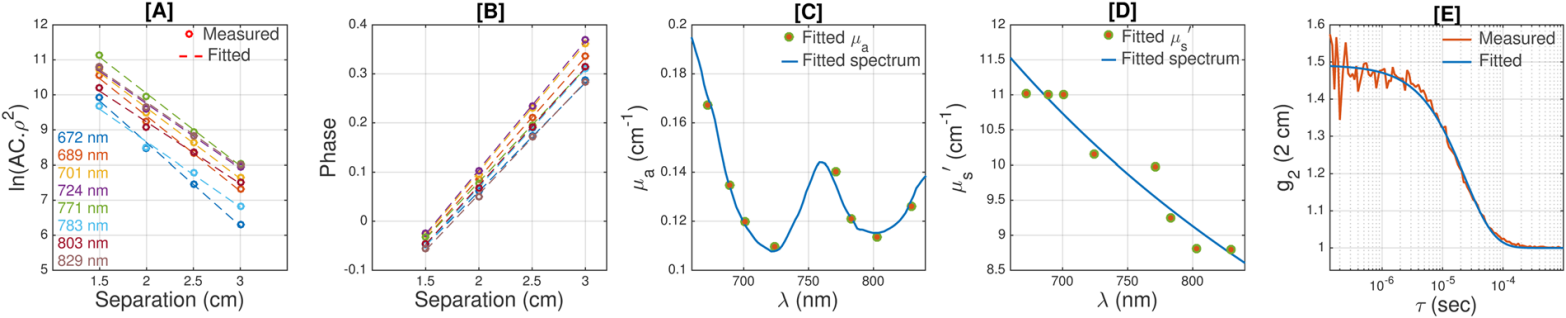
An example of fitting quality of the measured parameters. [**A**] The values of ln(*AC*.*ρ*^2^) as a function of source-detector distances (*ρ*) are presented by circles. Different colors correspond to different wavelengths (color-mapped in the legend) and the dashed lines are the corresponding linear fit. [**B**] Phase shift (radian) as a function of distance at the 8 wavelengths. [**C**] The circles are the measured absorption coefficients (*μ*_*a*_) at 8 wavelengths; the blue line is the fitted absorption spectra of oxy- and deoxy-hemoglobin. [**D**] The circles are the reduced scattering coefficients (${\mu }_{s}{\rm{^{\prime} }}$) at the 8 wavelengths and the blue line is the fitted scattering model. [**E**] The measured intensity autocorrelation function (red) measured with DCS and the fitted theoretical model (blue).

Figure 6 shows an example of the fitting quality of the measured parameters from a representative data set. Panels [A] and [B] present the values of ln(*AC*.*ρ*^2^) and phase as functions of source-detector distance (*ρ*), where reflected light’s amplitude (AC) and phase shift (Phase) at 4 source detector separations (1.5, 2.0, 2.5, and 3.0 cm) and at eight wavelengths (672, 689, 701, 724, 771, 783, 803, 829 nm) are measured by the FDNIRS system. One can calculate the wavelength-dependent tissue absorption and reduced scattering coefficient (*μ*_*a*_ and ${\mu }_{s}{\rm{^{\prime} }}$) using the slope of the linear fit of ln(*AC*.*ρ*^2^) and phase over source-detector separations^60^. To ensure the data quality, as previously done^43^, we discarded data for which the R-squared of the linear fit was smaller than 0.95. To measure the concentrations of oxy- and deoxy-hemoglobin, the hemoglobin absorption spectrum is fitted to the measured *μ*_*a*_ over all eight wavelengths (Fig. 6C), assuming a concentration of water of 75%^41,69^ to obtain concentration of oxy- and deoxy-hemoglobin (*C*_*Hbo*_, *C*_*HbR*_). Then, we can estimate the absorption coefficient at any desired wavelength by using following equation: ${\mu }_{a}(\lambda )={\varepsilon }_{Hbo}(\lambda ){C}_{Hbo}+{\varepsilon }_{HbR}(\lambda ){C}_{HbR}+0.75{\varepsilon }_{{H}_{2}O}$. Similarly, an inverse power law relationship derived from the Mie model^41^ was fitted to ${\mu }_{s}{\rm{^{\prime} }}$ over all eight wavelengths to measure effective particle size and number density (Fig. 6D), which enables us to calculate ${\mu }_{s}{\rm{^{\prime} }}$ at all desired wavelengths $({\mu }_{s}{\rm{^{\prime} }}(\lambda )=a{(\frac{\lambda }{500{\rm{n}}{\rm{m}}})}^{-b})$. Again, to ensure data quality, fits with p-values larger than 0.03 and R-squared smaller than 0.6 were discarded. Finally, the values of *μ*_*a*_ and ${\mu }_{s}{\rm{^{\prime} }}$ were extrapolated using the above relations to estimate *μ*_*a*_ and ${\mu }_{s}{\rm{^{\prime} }}$ at 850 nm, the operating wavelength of the DCS. Using the extrapolated *μ*_*a*_ and ${\mu }_{s}{\rm{^{\prime} }}$ in equation 1 and using the Siegert relation, we fitted the theoretical g_2_ to the measured intensity autocorrelation to obtain CBF_i_ (Fig. 6E).

### Statistical analysis

Each FDNIRS-DCS measurement lasted 10 seconds; FDNIRS data was obtained at 10 Hz and DCS autocorrelation curves were obtained at 1.2 Hz. We calculate the median of 100 FDNIRS data-points (amplitude and phase) at each separation/wavelength to increase the signal-to-noise ratio before performing the fitting for *μ*_*a*_(*λ*) and ${\mu }_{s}{\rm{^{\prime} }}$(*λ*). Similarly, for DCS, the fitting is performed on the average of the 12 intensity autocorrelation curves and the fitting output is reported as the CBF_i_ of that measurement. Since the number of infants of each group (male and female) is different, gestational ages are different and each group consists of several measurements on different sites (left, right, and middle), to investigate the statistical difference between male and female we have used a linear mixed effects (LME) model using the “fitlme” function of MATLAB. This model considers sex as the fixed effect and the variances of the unequal number of measurements in locations and lobes as well as the difference between the gestational age for each individual subject as the random effect. In order to investigate the effect of gestational age, we employed a similar LME approach with GA as the fixed effect and subjects, measurement side (left or right), lobes, and sex as random effects. Lastly, to compare the sexual differences in the demographic parameters such as infants’ weight, we have performed a two-sample Student’s t-test.
