# Publisher Correction: Shedding light on the neonatal brain: probing cerebral hemodynamics by diffuse optical spectroscopic methods

**DOI:** 10.1038/s41598-018-24115-6

**Published:** 2018-04-12

**Authors:** Parisa Farzam, Erin M. Buckley, Pei-Yi Lin, Katherine Hagan, P. Ellen Grant, Terrie Eleanor Inder, Stefan A. Carp, Maria Angela Franceschini

**Affiliations:** 1 Athinoula A. Martinos Center for Biomedical Imaging, Massachusetts General Hospital, Harvard Medical School, Boston, MA 02129 USA; 2 Georgia Institute of Technology, Atlanta, GA 30322 USA; 3 Fetal-Neonatal Neuroimaging and Developmental Science Center, Division of Newborn Medicine, Boston Children’s Hospital, Harvard Medical School, Boston, MA 02115 USA; 4 Department of Pediatric Newborn Medicine, Brigham and Women’s Hospital, Harvard Medical School, Boston, MA 02115 USA

Correction to: *Scientific Reports* 10.1038/s41598-017-15995-1, published online 17 November 2017

The competing financial interests statement in this Article should read:

“The authors hold patents on this technology.”

